# Usefulness of 99mTc-HMDP scintigraphy for the etiologic diagnosis and prognosis of cardiac amyloidosis

**DOI:** 10.1186/1750-1172-10-S1-P49

**Published:** 2015-11-02

**Authors:** Arnault Galat, Jean Rosso, Aziz Guellich, Axel Van Der Gucht, Jean-Luc Dubois-Randé, Violaine Plante-Bordeneuve, Emmanuel Itti, Thibaud Damy

**Affiliations:** 1 CHU H Mondor, Amyloidosis Mondor Creteil, 94000, Créteil, France

## Background

Amyloidosis is characterized by extracellular deposits of insoluble proteins that cause tissue damage. The three main types are monoclonal light chain (AL), wild-type transthyretin (wt-TTR), and mutated transthyretin (m-TTR) amyloidosis. Cardiac amyloidosis (CA) raises diagnostic challenges.

## Objective

To assess the diagnostic accuracy of 99mTc-HMDP-scintigraphy for typing CA, differentiating CA from non-amyloid left ventricle hypertrophy (LVH), and predicting outcomes.

## Methods

121 patients with suspected CA underwent 99mTc-HMDP-scintigraphy in addition to standard investigations.

## Results

CA was diagnosed in all AL (n=14) and wt-TTR (n=21). Among m-TTR (n=34), 26 had CA, 4 neuropathy without CA and 4 were asymptomatic carriers. Of the 52 patients with non-amyloid heart disease, 37 had LVH and served as controls. 99mTc-HMDP cardiac uptake occurred in all wt-TTR, in m-TTR with CA except two, and in one AL. A visual score ≥2 was 100% specific for diagnosing TTR-CA. Among TTR-CA, heart-to-skull retention (HR/SR) correlated with CA severity (LVEF and NT-proBNP). Median follow-up was 111 days (50;343). In a multivariate Cox model including clinical, echocardiographic, and scintigraphic variables, NYHA III-IV and HR/SR>1.94 predicted acute heart failure and/or death.

## Conclusions

99mTc-HMDP-scintigraphy allows differentiating transthyretin from AL-CA and CA from other LVHs and also provides prognostic information.

